# ceRNAshiny: An Interactive R/Shiny App for Identification and Analysis of ceRNA Regulation

**DOI:** 10.3389/fmolb.2022.865408

**Published:** 2022-05-13

**Authors:** Yueqiang Song, Jia Li, Yiming Mao, Xi Zhang

**Affiliations:** ^1^ State Key Laboratory of Genetic Engineering, School of Life Sciences, Fudan University, Shanghai, China; ^2^ Department of Thoracic Surgery, Suzhou Kowloon Hospital, School of Medicine, Shanghai Jiao Tong University, Suzhou, China; ^3^ Department of Rehabilitation, Huashan Hospital, Fudan University, Shanghai, China

**Keywords:** lncRNA, miRNA, ceRNA, R, Shiny

## Abstract

The competing endogenous RNA (ceRNA) network is a newly discovered post-transcriptional regulation that controls both physiological and pathological progresses. Increasing research studies have been pivoted on this theory to explore the function of novel non-coding RNAs, pseudogenes, circular RNAs, and messenger RNAs. Although there are several R packages or computational tools to analyze ceRNA networks, an urgent need for easy-to-use computational tools still remains to identify ceRNA regulation. Besides, the conventional tools were mainly devoted to investigating ceRNAs in malignancies instead of those in neurodegenerative diseases. To fill this gap, we developed ceRNAshiny, an interactive R/Shiny application, which integrates widely used computational methods and databases to provide and visualize the construction and analysis of the ceRNA network, including differential gene analysis and functional annotation. In addition, demo data in ceRNAshiny could provide ceRNA network analyses about neurodegenerative diseases such as Parkinson’s disease. Overall, ceRNAshiny is a user-friendly application that benefits all researchers, especially those who lack an established bioinformatic pipeline and are interested in studying ceRNA networks.

## Introduction

The vast majority of the human genome is non-coding sequences whose transcripts without protein-coding capacity are named non-coding RNAs (ncRNAs) (Alexander et al., 2010). ncRNAs are now regarded as core regulators involved in gene transcription, epigenetic regulation, and post-transcriptional regulation which exert their effects on the occurrence, development, and diagnosis of diverse diseases (Esteller 2011). A hypothesis of how ncRNAs work has been proposed and gradually confirmed, which was named the competing endogenous RNAs (ceRNAs) network (Salmena et al., 2011). According to this theory, long non-coding RNAs (lncRNAs), pseudogenes, and circular RNAs (circRNAs) act as microRNA (miRNA) sponges via miRNA response elements (MREs) or messenger RNA (mRNA) binding sites to control the availability of endogenous miRNAs for binding to their target mRNAs, which can form a ceRNA network to modulate mRNA expression and regulate protein levels. These complex networks may provide multiple clues for unraveling the pathogenesis in diseases (Tay et al., 2014). Notably, as a representative of various types of ceRNAs, lncRNA-associated ceRNA networks might be eligible candidates as promising therapeutic targets.

Due to the huge scale of ceRNA networks, the availability of computational methods has allowed theoretical construction of ceRNA networks which provides convincing evidence for further verification *in vitro* or *in vivo* (Yang et al., 2018). The development of different ceRNA-directed computational methods can be mainly categorized into two classes: 1) methods constructed by combining expression profiles and statistic indexes, such as the Pearson correlation coefficient (PCC) and mutual information (MI), sensitivity correlation (SI), multiple sensitivity correlation, conditional mutual information (CMI), intervention calculus when the DAG is absent (IDA), and liquid association (LA) (Lloyd 2000), and 2) mathematical models, such as the minimal model, stochastic model, mass-action model, coarse-grained model, and coarse-grained competition motif model (Le et al., 2017; Zhang et al., 2017, 2022). Additionally, a set of lncRNA–miRNA–mRNA pairs databases have been established (Li et al., 2014; Le et al., 2017), such as lnCeDB (Das et al., 2014), LncCeRBase (Pan et al., 2019), miRSponge (Wang et al., 2015), LncACTdb (Wang et al., 2022), ceRDB (Sarver Subramanian 2012), starBase (Li et al., 2014), HumanViCe (Ghosal et al., 2014), PceRBase (Yuan et al., 2017), Tarbase (Vergoulis et al., 2012), miRTarbase (Huang et al., 2020), miRecords (Xiao et al., 2009), miRWalk (Sticht et al., 2018), TargetScan (www.targetscan.org), miRanda (Betel et al., 2008), MicroCosm (Griffiths-Jones et al., 2008), PicTar (http://www.pictar.org/), DIANA-microT (Vlachos et al., 2015), PITA (http://genie.weizmann.ac.il/pubs/mir07/mir07_data.html), and CLASH (Helwak Tollervey 2014). Some databases can function as not only a prediction tool to guide experiments but also a free hub which provides experimentally validated results.

Current computational methods are, respectively, biased in terms of the accuracy or sensitivity of prediction. The contents of different databases also vary with species, diseases, and tissues. At present, scholars have developed many R packages for constructing ceRNA networks, but all of them require proficiency in using R software (Li et al., 2018; Zhang et al., 2018, 2019, 2021; Wen et al., 2020). Meanwhile, the newly developed CeNet Omnibus, an R/Shiny-based application, is used to predict ceRNA network construction using different computational methods (Wen et al., 2021) with database information uploaded manually, which also analyzes the distribution of topological properties of networks. Then, ceRNAshiny, which we developed, has the advantage of being more convenient and reliable for students and researchers who majored in biology and medicine, especially people with limited experience in programing. Analysis processes in ceRNAshiny for sequencing data are currently recognized and meet basic requirements of users for sequencing data analysis. At the same time, ceRNAshiny can effectively classify large sequencing data by RNA types, which is important and convenient for distinguishing sequencing data containing various RNA types. In addition, ceRNAshiny provides predictions based on expression, sequence, and both. In terms of built-in databases, ceRNAshiny includes predictive and experimentally validated databases that, respectively, contain the human-sourced contents of multiple databases. Based on these two types of databases, users can obtain multiple ceRNA networks more easily, providing multiple options for subsequent data validation and wet experiments. Overall, ceRNAshiny could be a useful tool for people without enough time and a knowledge background to rapidly get results. With the support of the R/Shiny framework, ceRNAshiny offers a web-based user-friendly interface for users to obtain the identification, analysis, and visualization of ceRNA regulation, such as differential gene analysis and functional annotation.

## Implementation

ceRNAshiny is an R-based Shiny application constructed using various R packages, including reshape2 (https://rdocumentation.org/packages/reshape2/versions/1.4.3), igraph (Csardi Nepusz 2006), edgeR (Robinson et al., 2010), DESeq2 (Michael I Love and Anders, 2014), limma (Brophy et al., 1987; Ritchie et al., 2015), glmnet (Simon et al., 2011; Engebretsen and Bohlin 2019), yulab. utils (https://CRAN.R-project.org/package=yulab.utils), ggplot2 (Wickham et a.l, 2016), rvcheck (https://github.com/GuangchuangYu/rvcheck), shiny (https://github.com/rstudio/shiny/issues), shinythemes (https://rstudio.github.io/shinythemes/), DT (https://github.com/rstudio/DT), pheatmap (https://CRAN.R-project.org/package=pheatmap), ReactomeRA (Yu and He 2016), and clusterProfiler (Yu et al., 2012). The Shiny R platform was deployed on the webserver to host the web application of ceRNAshiny. The human-sourced lncRNA–miRNA–mRNA pairing information included in ceRNAshiny was obtained from various databases (John et al., 2004; Lewis et al., 2005), which could fall into two categories: predicted databases, like starBase 3.0 (starBase 3.0 was also named as ENCORI; Li et al., 2014), miRWalk (Sticht et al., 2018), TargetScan (www.targetscan.org), and miRanda (Betel et al., 2008), and experimentally validated databases, like Tarbase (Vergoulis et al., 2012) and miRTarbase (Huang et al., 2020). To show the functionality and usability of ceRNAshiny, we used the publicly available array datasets (GSE7621) that were generated from the substantia nigra from the postmortem human brain of Parkinson’s disease (PD) patients and control ones (Lesnick et al., 2007). Furthermore, GSE136666, which contains transcriptomic results of human substantia nigra and putamen samples from PD patients and age-matched controls, was chosen as template data of high-throughput RNA sequencing data (Xicoy et al., 2020). The app can be accessed here: https://cerna.shinyapps.io/cerna_shiny/. The source code and related documents can be obtained through GitHub: https://github.com/yqsongGitHub/ceRNA_shiny.

## Data Input

The ceRNAshiny app is more suitable for users who are interested in ceRNA but are limited by programing. For an individual user, the only step is to input the expression matrix, group list, and annotation platform information (optional) that are consistent with the format of template data (could be downloaded). Both array data and high-throughput RNA sequencing data can be acceptable and processible as the input expression matrix. If the input expression matrix is array data, it is necessary to synchronously provide annotation platform information, with probe names as row names of the matrix and sample lists as column names. In case the input expression matrix comprised high-throughput RNA sequencing data, Ensembl numbers should be provided as row names and sample lists as column names.

## Data Processing and Analysis

For data processing, the following parameters are performed, including missing value imputation, log2 transformation, background adjustment, and quantile normalization. Cluster analysis results are presented in heat maps to allow users to remove low-quality samples. Depending on the category of input data, differential gene analysis, annotation analysis, and enrichment analysis can be performed using the corresponding R packages.

## Network Construction

Based on differentially expressed genes (DEGs), PCC, sensitivity partial Pearson correlation (SPPC), and the partial Pearson correlation (PC) algorithm are supposed to be employed for predicting potential lncRNA–miRNA–mRNA pairs, which can identify the maximum number of miRNA sponge interactions (Zhang et al., 2019). In addition, we downloaded and compared the relevant information in starBase 3.0 (Li et al., 2014), miRWalk (Sticht et al., 2018), TargetScan (www.targetscan.org), and miRanda (Betel et al., 2008) databases and aggregated miRNA–lncRNA pairs (63,556 pairs) and miRNA–mRNA pairs (1,441,765 pairs) recorded in these databases as the predicted database. For the experimentally validated database, we aggregated the contents of the Tarbase database (Vergoulis et al., 2012) and miRTarbase database (Huang et al., 2020), which finally contained miRNA–lncRNA pairs (1,506 pairs) and miRNA–mRNA pairs (652,703 pairs). Thus, we predicted ceRNA networks using three approaches. In the first approach, we analyzed statistical relationships of genes based on gene expression and different algorithms (PCC, SPPC, and PC) to identify potential lncRNA–miRNA–mRNA pairs. In the second approach, we compared uploaded data with the predicted database and the experimentally verified database, respectively, to get the potential lncRNA–miRNA–mRNA pairs based on the sequence. Then in the last approach, we intersected the results of the previous two steps to obtain lncRNA–miRNA–mRNA pairs that satisfied both requirements. Then the intersection of the aforementioned results would be output as more credible clues for subsequent experiments. Finally, enrichment analysis is going to be performed on the aforementioned ceRNA networks for biological functions and pathways.

## Results

### Case Study: GSE7621 Expression Data of Substantia Nigra from a Postmortem Human Brain of Parkinson’s Disease

For demonstration, we used the expression profiling dataset GSE7621 from the GPL570 [HG-U133_Plus_2] Affymetrix Human Genome U133 Plus 2.0 Array platform (54317 probes per sample) to identify ceRNA networks in 25 cases with PD (Lesnick et al., 2007). In addition, the dataset GSE13666 from GPL11154 Illumina HiSeq 2000 (*Homo sapiens*) was chosen as the template data of high-throughput RNA sequencing data (Xicoy et al., 2020). Due to the similar process, the analysis process of GSE7621 is selected as the example.

**FIGURE 1:**
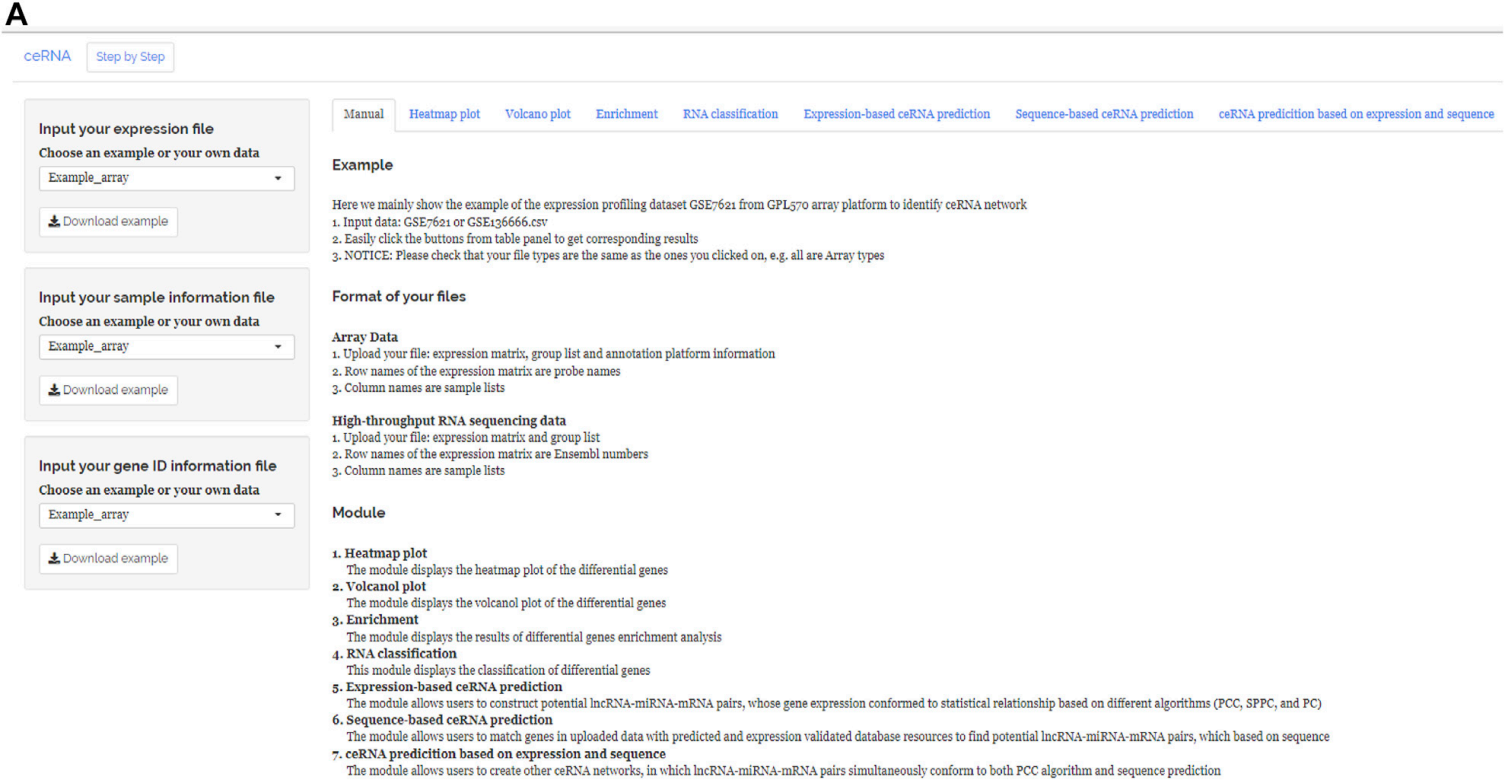
Main interface of ceRNAshiny. **(A)** The main interface of ceRNAshiny for introduction and analysis.

**FIGURE 2:**
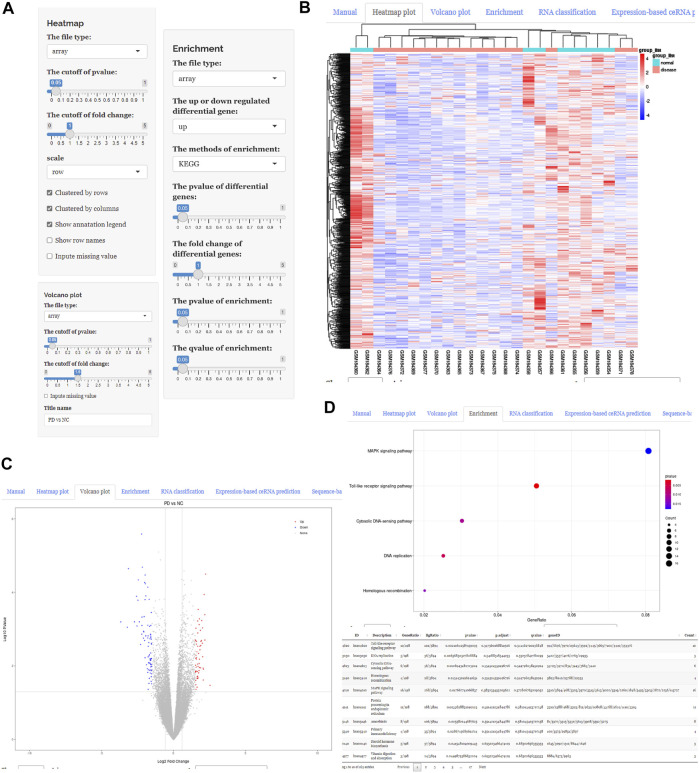
Using ceRNAshiny to generate differentially expressed genes and enrichment analysis. **(A)** Panels for parameter configuration. **(B)** The generated heatmap plot using the template dataset. **(C)** The generated volcano plot using the template dataset. **(D)** The generated enrichment analysis using the template dataset.

**FIGURE 3:**
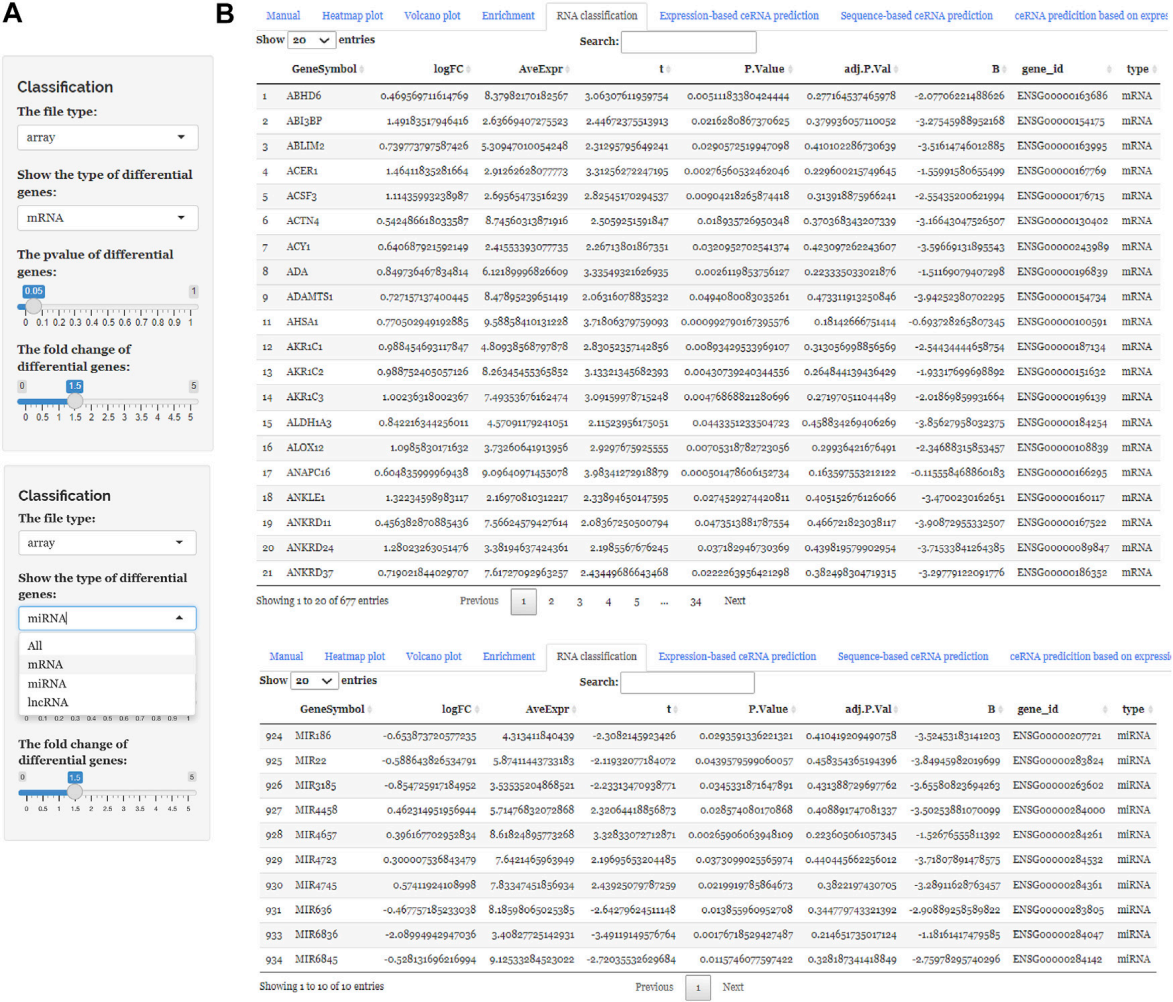
Using ceRNAshiny to classify RNAs. **(A)** Panels to show the type of differential genes and to adjust parameters. **(B)** The generated results of RNA classification using the template dataset.

After uploading the expression profiling data into the input table panel of ceRNAshiny (Figure 1A), data can be imported conveniently for further analyses. Through easily clicking the buttons from the table panel, users can perform the following editing: “Heatmap plot,” “Volcano plot,” “Enrichment,” “RNA classification,” “Expression-based ceRNA prediction,” “Sequence-based ceRNA prediction,” and “ceRNA prediction based on expression and sequence” (Figure 1A). The analyses indicated by these buttons are based on differential gene analysis. Sliders of the *p* value and fold change value are set up for users to adjust the *p* value and fold change value independently and to gain corresponding plots (volcano plot, heatmap plot, enrichment plots, etc.) and results (Figure 2). Moreover, ceRNAshiny can help users effectively distinguish lncRNAs, miRNAs, and mRNAs among multitudinous genes in the expression profiles (Figure 3A). It is worth noting that the correct type of uploaded data should be selected in the panel to avoid errors during these analyses (Figure 3B).

**FIGURE 4:**
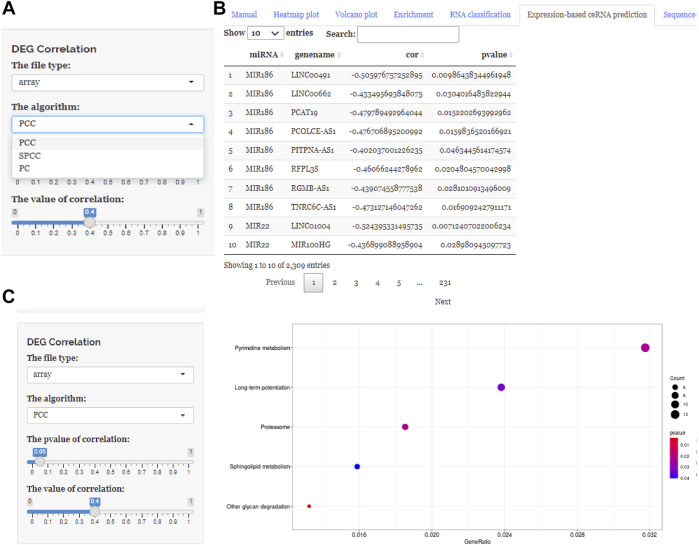
Using ceRNAshiny to generate “Expression-based ceRNA prediction.” **(A)** Panels to show the type of arithmetic and adjust parameters. **(B)** The generated algorithm prediction and its enrichment results based on PCC, PC, and SPPC arithmetic using the template dataset.

**FIGURE 5:**
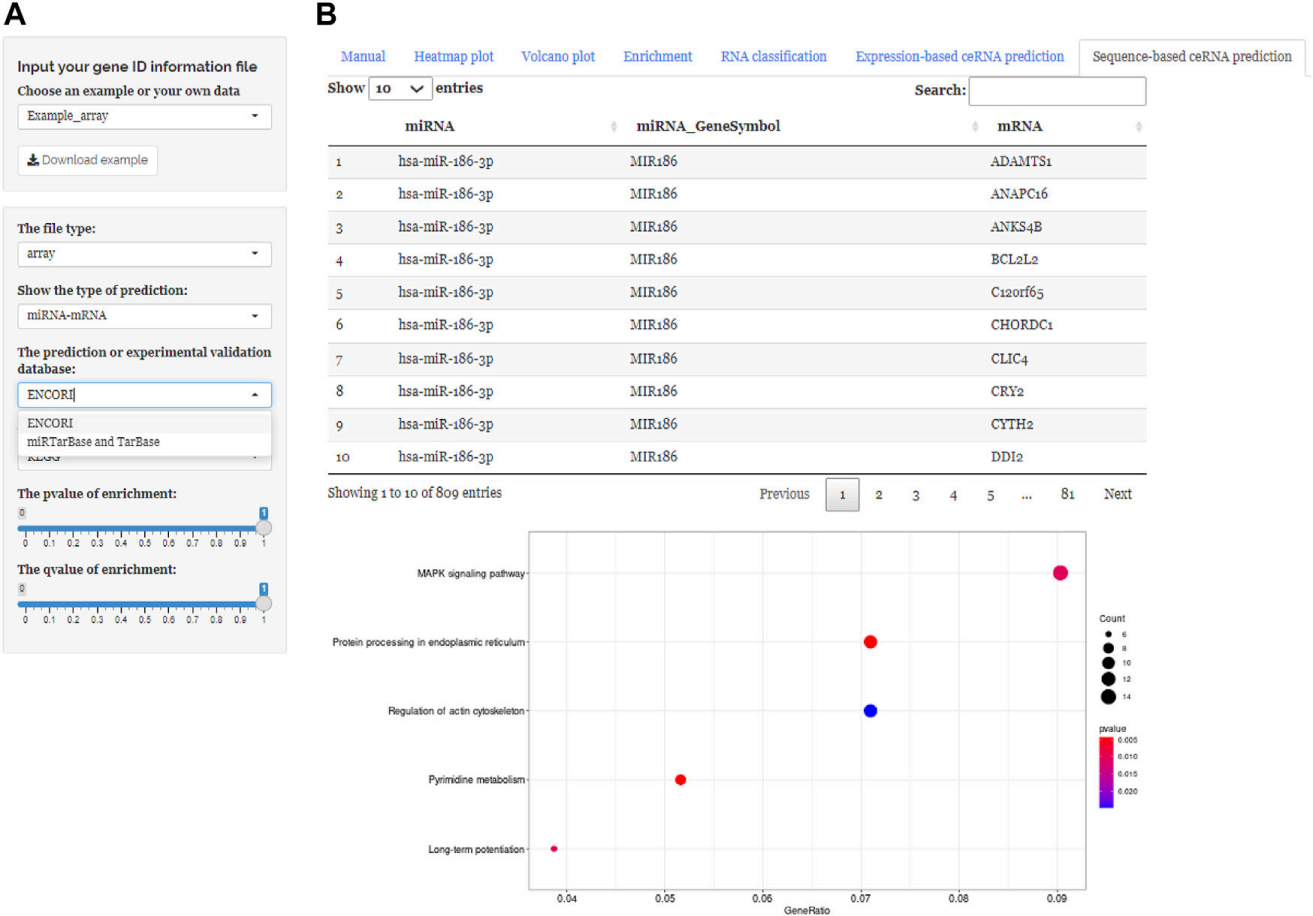
Using ceRNAshiny to generate “Sequence-based ceRNA prediction.” **(A)** Panels to adjust parameters. **(B)** The generated database prediction and its enrichment results based on databases using the template dataset.

**FIGURE 6:**
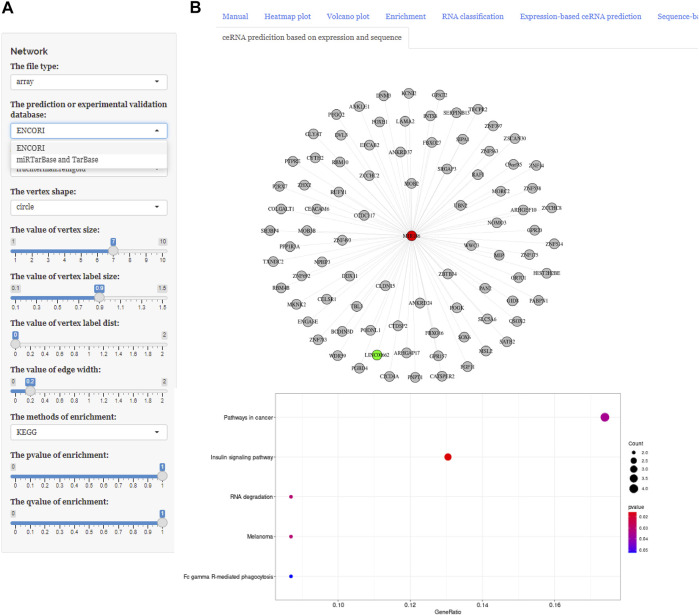
Using ceRNAshiny to generate “ceRNA prediction based on expression and sequence.” **(A)** Panels to adjust parameters. **(B)** The generated ceRNA network and its enrichment results based on PCC arithmetic and database sources using the template dataset.

These three buttons in the interface, “Expression-based ceRNA prediction,” “Sequence-based ceRNA prediction,” and “ceRNA prediction based on expression and sequence,” are designed for identifying ceRNA networks. Users can select the “Expression-based ceRNA prediction” module to construct and, respectively, download potential lncRNA–miRNA–mRNA pairs, whose gene expression conformed to statistical relationships based on different algorithms (PCC, SPPC, and PC) (Figures 4A–C). In addition, the “Sequence-based ceRNA prediction” module allows users to match genes in uploaded data with predicted and expression validated database resources to find potential lncRNA–miRNA–mRNA pairs, based on sequence (Figures 5A, B). Finally, through the “ceRNA prediction based on expression and sequence” module, users can create other ceRNA networks, in which lncRNA–miRNA–mRNA pairs simultaneously conform to both the PCC algorithm and sequence prediction (Figures 6A, B). The analysis is based on default parameters, which can be adjusted in accordance to requirements.

## Conclusion and Outlook

ceRNAshiny is designed with a clear purpose to identify and visualize ceRNA networks for users with limited experience in programing. ceRNAshiny was developed for customizable generation of volcano plots, heatmap plots, ceRNA graphs, and other results using input expression datasets. Unlike conventional tools, ceRNAshiny not only identified the ceRNA networks using computational methods but also matched lncRNA–miRNA–mRNA pairs from multiple human source databases, eliminating the need for users to use online databases. With the basic R environment and an internet connection, this user-friendly Shiny application will be automatically set up and intuitively applied to visually review the reported results. We provide a downloadable source code and an offline version for researchers who are good at programming. Since the Shiny package was designed to build interactive web applications, it is straightforward to deploy ceRNAshiny on servers to provide an online service so that it can be used by researchers from a variety of backgrounds with ranging interests.

## Data Availability

The original contributions presented in the study are included in the article/Supplementary Material; further inquiries can be directed to the corresponding authors.
